# Case report: Metastatic refractory undifferentiated small round-cell sarcoma successfully treated with surufatinib and camrelizumab

**DOI:** 10.3389/fonc.2024.1416241

**Published:** 2024-07-11

**Authors:** Yong Li, Jinpeng Huang, Xian Chen, Yongsong Ye, Xiaohua Du, Ioannis A. Voutsadakis, Mahesh Seetharam, Haibo Zhang, Min Lu

**Affiliations:** ^1^ Department of Oncology, The Second Affiliated Hospital of Guangzhou University of Chinese Medicine, Guangzhou, China; ^2^ Department of Oncology, Guangdong Provincial Hospital of Traditional Chinese Medicine, Guangzhou, China; ^3^ Department of Oncology, Guangdong Provincial Hospital of Chinese Medicine, Zhuhai, China; ^4^ Department of Image, Guangdong Provincial Hospital of Chinese Medicine, Guangzhou, China; ^5^ Department of Pathology, Guangdong Provincial Hospital of Chinese Medicine, Guangzhou, China; ^6^ Division of Medical Oncology, Sault Area Hospital, Sault Ste Marie, ON, Canada; ^7^ Division of Clinical Sciences, Section of Internal Medicine, Northern Ontario School of Medicine, Sudbury, ON, Canada; ^8^ Department of Hematology and Oncology, Mayo Clinic, Phoenix, AZ, United States

**Keywords:** undifferentiated small round-cell sarcoma, surufatinib, programmed death-1 inhibitor, target therapy, case report

## Abstract

**Background:**

Undifferentiated small round-cell sarcomas (uSRCSs) are a subgroup of sarcomas that are difficult to diagnose. Some uSRCSs have specific gene re-arrangements, but others do not. Currently, there is no specific treatments for advanced uSRCSs, and its treatment is largely based on general experience with sarcomas, which includes chemotherapy, targeted therapy, and immunotherapy. In this article, we report a patient with uSRCS who responded to treatment with anti-VEGF inhibitor surufatinib and anti-PD-1 inhibitor camrelizumab after progression on first-line chemotherapy, second-line anlotinib combined with immunotherapy, and third-line chemotherapy.

**Case description:**

In July 2020, a 37-year-old female patient was diagnosed with advanced uSRCS. Results for the Ewing sarcoma RNA binding protein 1 and Wilms tumor suppressor (EWSR1/WT1) fusion gene were negative. The patient was also negative with BCOR (BCL6 co-repressor) and CIC (capicua transcriptional repressor) fusion gene. The next-generation sequencing results revealed point mutations on Phosphatidylinositol-4,5-Bisphosphate 3-Kinase Catalytic Subunit Beta (PIK3CB), Transcription Factor Binding To IGHM Enhancer 3 (TFE3), Mucin 16 (MUC16), and AXL (Axl, also called UFO, ARK, and Tyro7, is part of a family of receptor tyrosine kinases). The patient received 4 cycles of the Ifosfamide and epirubicin hydrochloride regimen, and her best objective response was stable disease. On November 3, 2020, a computed tomography (CT) scan revealed progressive disease (PD). Two cycles of camrelizumab (a programmed death-1 inhibitor) plus anlotinib (an anti- vascular endothelial growth factor drug) were administered, but PD was again observed. Thus, a regimen of gemcitabine plus docetaxel was adopted. Unfortunately, the disease progressed once again after two cycles of the treatment. On February 4, 2021, the patient began to receive targeted therapy with surufatinib combined with camrelizumab. A CT scan showed that the tumor achieved a partial response. As of April 2023, the patient had a progression-free survival time of 26 months.

**Conclusions:**

Surufatinib in combination with camrelizumab could be effective in the treatment of advanced uSRCSs.

## 1 Introduction

Undifferentiated small round-cell sarcomas (uSRCSs) are a subgroup of sarcomas and are defined by the World Health Organization (WHO, 2020) as soft tissue and bone tumors (1). USRCSs are difficult to identify because of their undifferentiated round cytomorphology and scant stromal changes, and are mostly high-grade tumors. Ewing sarcoma RNA binding protein 1 (*EWSR1)* gene fusion, Capicua transcriptional repressor gene(CIC)-rearranged genes, and BCL6 corepressor (BCOR)-rearranged genes are usually tested to clarify their genetic types, which include Ewing sarcomas, round-cell sarcomas with EWSR1 gene fusion and non-ETS fusion, CIC-rearranged sarcomas, and BCOR-rearranged sarcomas. However, some uSRCS do not show specific gene fusions.

Advanced uSRCSs comprise a heterogeneous group of highly aggressive tumors associated with a poor prognosis, especially in metastatic disease which are treated according to the conventional recommended regimens for sarcomas, which include chemotherapy using anthracyclines, alkylating agents, topoisomerase inhibitors, gemcitabine and docetaxel (2, 3). Novel targeted drugs have been approved, including pazopanib, regorafenib, and anlotinib (which was approved in China) for second or later line therapies (4–6). Although it has not yet been approved for this indication, immunotherapy has shown promise in some subtypes of sarcoma (7). However, currently there is a paucity of efficacious regimens after the third-line treatment.

Surufatinib is a multi-target drug that has the potential to reverse resistance to the dominant single anti-VEGF anlotinib. In this article, we report a case of a patient with uSRCS who responded to treatment with surufatinib and camrelizumab after resistance to first-line chemotherapy, second-line anlotinib combined with immunotherapy, and third-line chemotherapy. It is the first case report in which surufatinib was shown to be effective in treating uSRCS. Based on our results, we suggested that surufatinib could be effective in the treatment of some advanced uSRCSs in combination with immunotherapy.

## 2 Case description

**Figure 1:**
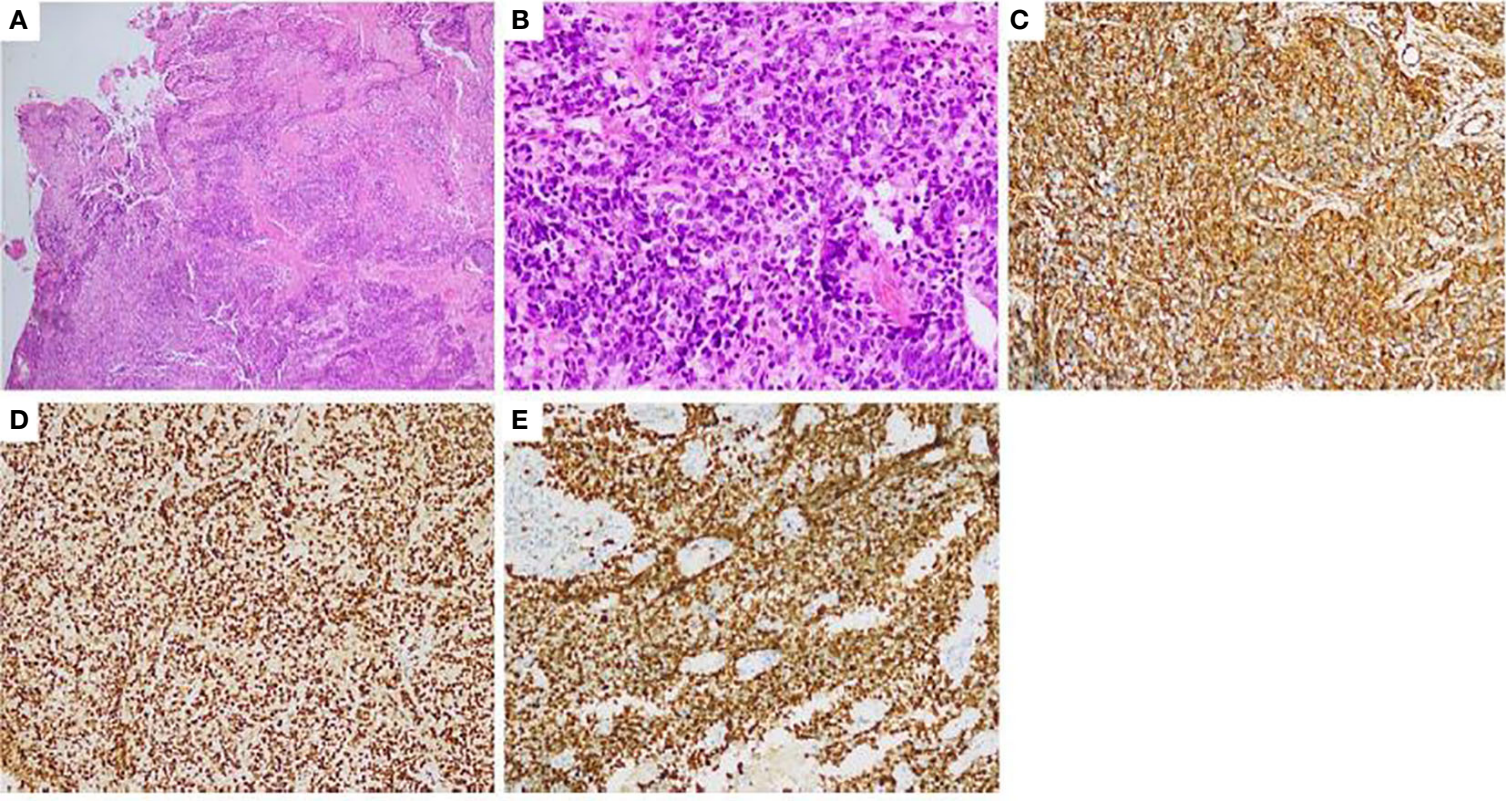
Pathology and immunohistochemistry of a left inguinal lymph node biopsy **(A)** HE 40x:the tumors appear to be arranged in sheets or nests; **(B)** HE 400x:The cytoplasm of tumor cells is sparse, the cell boundary is unclear. The round or oval nuclei and nuclear hyperchromatism and fission can be seen. immunohistochemical staining: **(C)** Vimentin: Diffuse cytoplasmic positive of tumor cells (HE 400x.); **(D)** INI-1: Nucleus positive (HE 400x.); **(E)** Ki67: It shows high proliferation of the tumor (400x.). HE, Hematoxylin-eosin stain.

In July 2020, a 37 years-old woman with no relevant past medical history, presented with left lumbar spine pain accompanied by mobility limitations including local edema and claudication for more than half a year, worsening over the last 2 months with analgesics. A positron emission tomography–computed tomography (PET-CT) scan was performed and showed multiple enlarged retroperitoneal lymph nodes and multiple masses in the vicinity of the left ischial tuberosity, left pubic bone, and left iliac acetabulum. Magnetic resonance imaging (MRI) showed left acetabular bone destruction which were considered bone metastases. A left inguinal lymph node biopsy was performed on July 15 2020. The pathological diagnosis was uSRCS and the immunohistochemical results were as follows: Vimentin (Vim) (+++), B-cell lymphoma 2(Bcl-2) (++), Desmin (+), Neuron-specific enolase (NSE) (++), Cluster of Differentiation 56(CD56) (+++), Synaptophysin (Syn) (+), Chromogranin A (CgA) (–), Cytokeratin AE1/AE3(CK AE1/AE3) (+), CK8 (positive in few cells), Cytokeratin 18(CK18) (positive in few cells), Myogenic differentiation 1(MyoD1) (positive in few cells), Myogenin (–), S100 protein (S100) (–), Human Melanoma Black 45(HMB45) (–), Melan-A (–), Cluster of Differentiation 20(CD20) (–), Cluster of Differentiation 3 (CD3) (–), Cluster of Differentiation 21(CD21) (–), P40 protein (P40) (–), Protein 16(P16) (–), Epithelial membrane antigen(EMA) (–), Thyroid transcription factor 1(TTF-1) (–), Cluster of Differentiation 99(CD99) (–), Friend leukemia integration 1(Fli-1) (–), Cluster of Differentiation 30(CD30) (–), Integrase interactor 1(INI-1) (+), and Ki67 antigen (Ki67) (90%+) (**Figure 1**). The Epstein-Barr virus-encoded small RNA (EBER) *in-situ* hybridization result was negative. PD-L1 staining was negative. A diagnosis of desmoplastic small round-cell tumor was excluded because the fluorescence *in-situ* hybridization assay result for the EWSR1/WT1 fusion gene was negative. The next-generation sequencing (NGS) results revealed PIK3CB, TFE3, MUC16 and AXL point mutations, but no other mutations were found. NGS of RNAseq fusion genes, including BCOR and CIC fusion gene, were negative (**Figure 1**).

**Figure 2:**
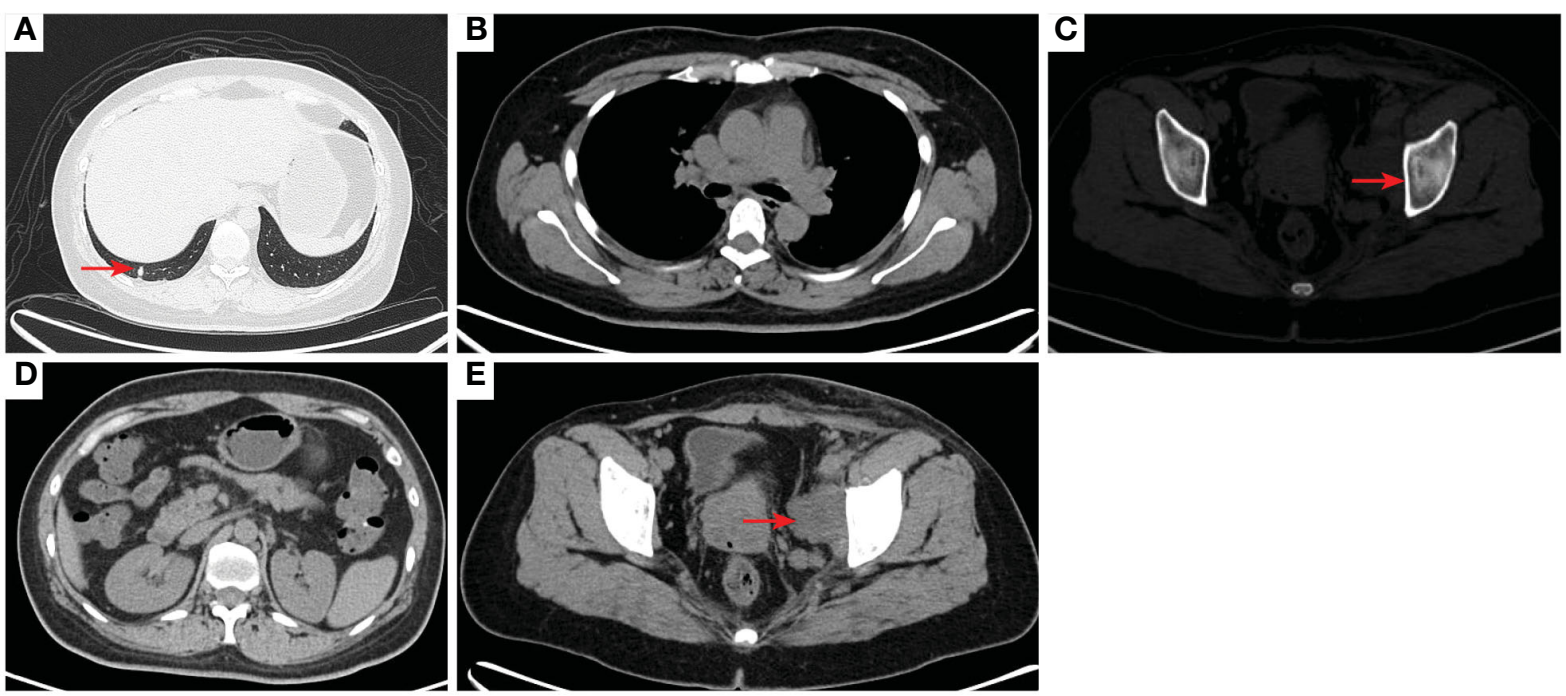
2020–7-10 CT scan: the metastatic tumor in the right lower lobe of the lung and the long diameter was 0.69 cm [red arrow, **(A)**]. Mediastinal lymph nodes were not significantly enlarged **(B)**. Left acetabular metastases [red arrow, **(C)**]. Abdominal and abdominal lymph nodes were not significantly enlarged **(D)** No enlarged lymph nodes were found in the right hilum and the retroperitoneal area. The left pelvic lymph nodes were found and the short diameter was 4.05cm [red arrow, **(E)**].

**Figure 3:**
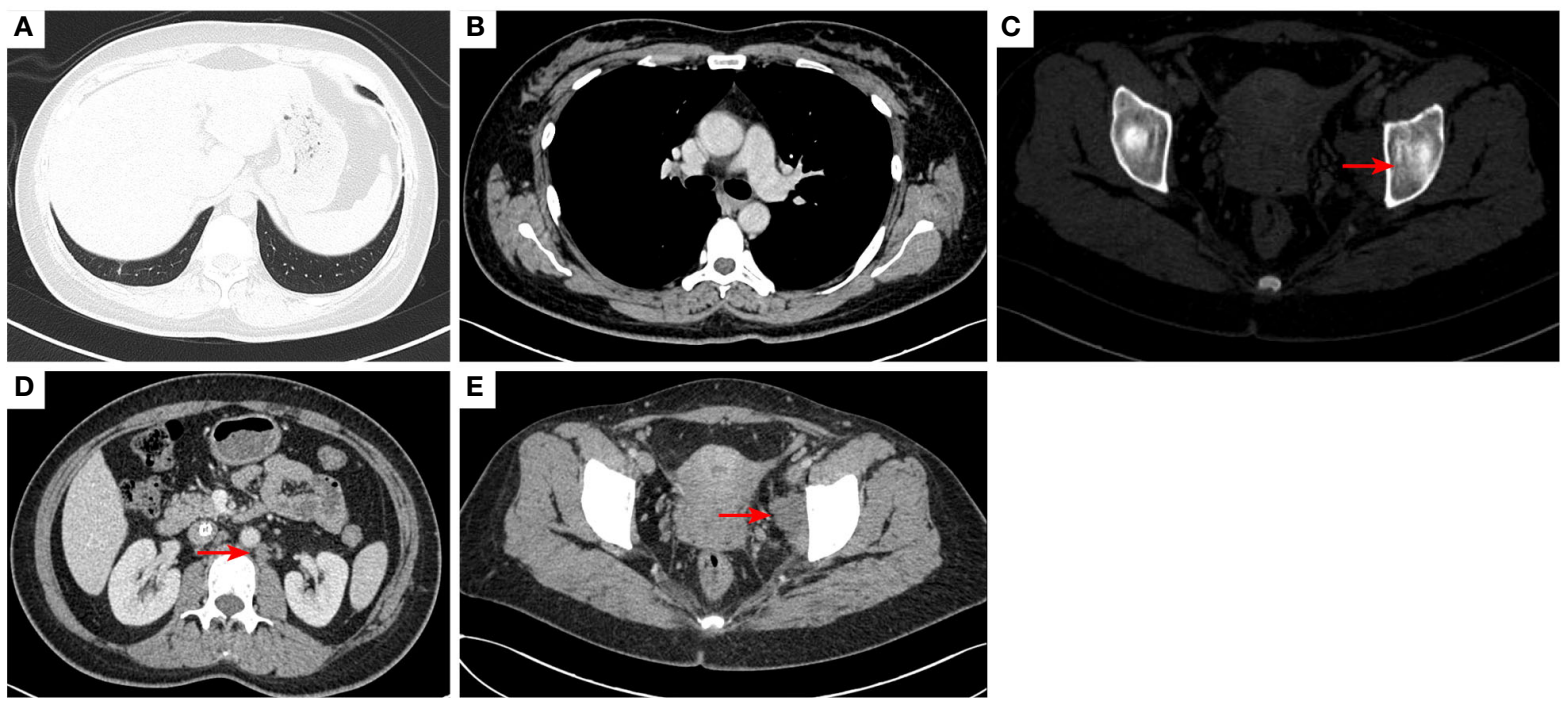
2020–9-16 CT scan: The original metastatic tumor in the right lower lobe of the lung disappeared **(A)**; no enlarged lymph nodes were found in the right hilum **(B)**. Left acetabular metastases [red arrow, **(C)**]. The short diameter of retroperitoneal lymph nodes was 0.91 cm which was a non-confirmed metastases [red arrow, **(D)**], and the short diameter of left pelvic lymph nodes was 2.98 cm [red arrow, **(E)**]. The efficacy was SD. SD, stable disease.

**Figure 4:**
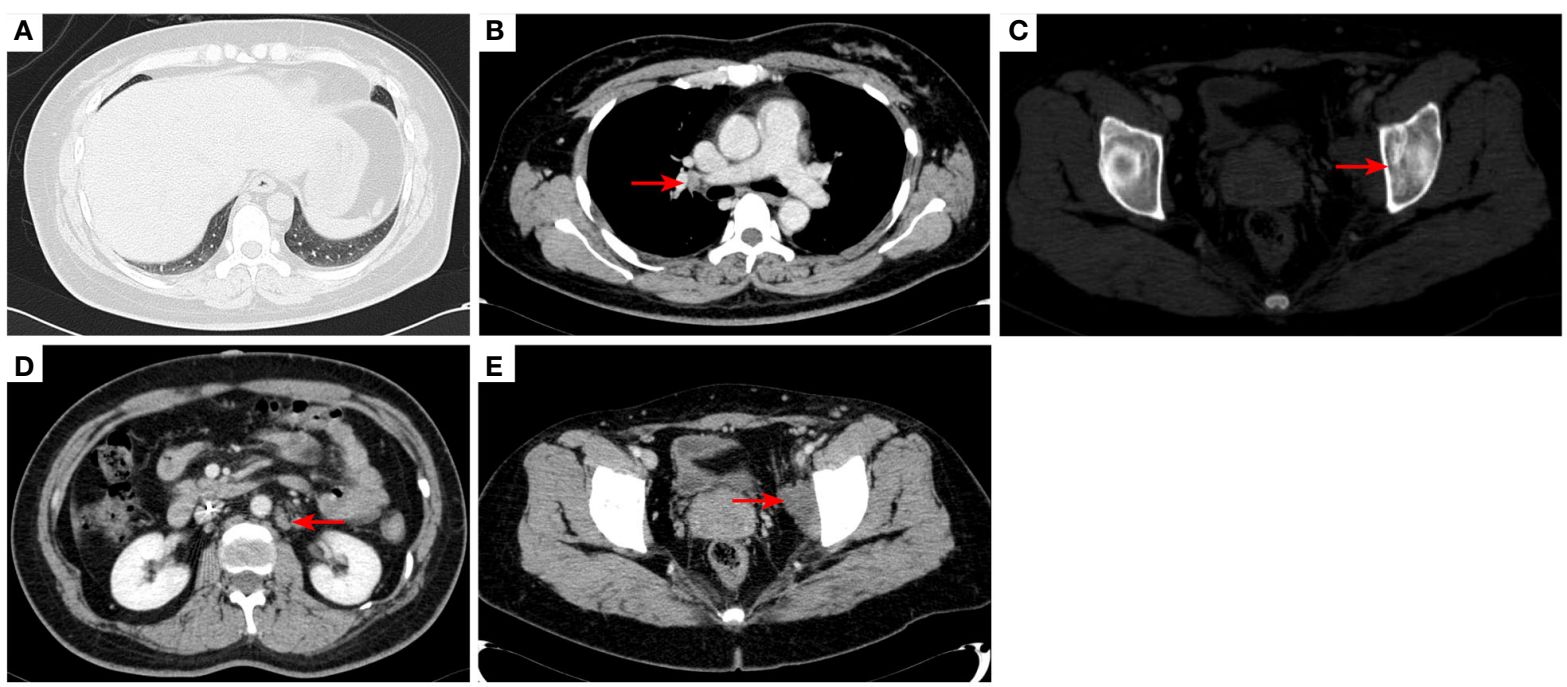
2020–11-3 CT scan: The original right lower lobe of the lung metastatic tumor disappeared **(A)**; short diameter of new right hilar lymph node was 0.83 cm [red arrow, **(B)**]; Left acetabular metastases (red arrow, **(C)**). The short diameter of retroperitoneal lymph nodes was 1.01 cm which was bigger than before [red arrow, **(D)**], and the short diameter of left pelvic lymph nodes was 2.76 cm [red arrow, **(E)**]. The efficacy was PD. PD, progressive disease.

**Figure 5:**
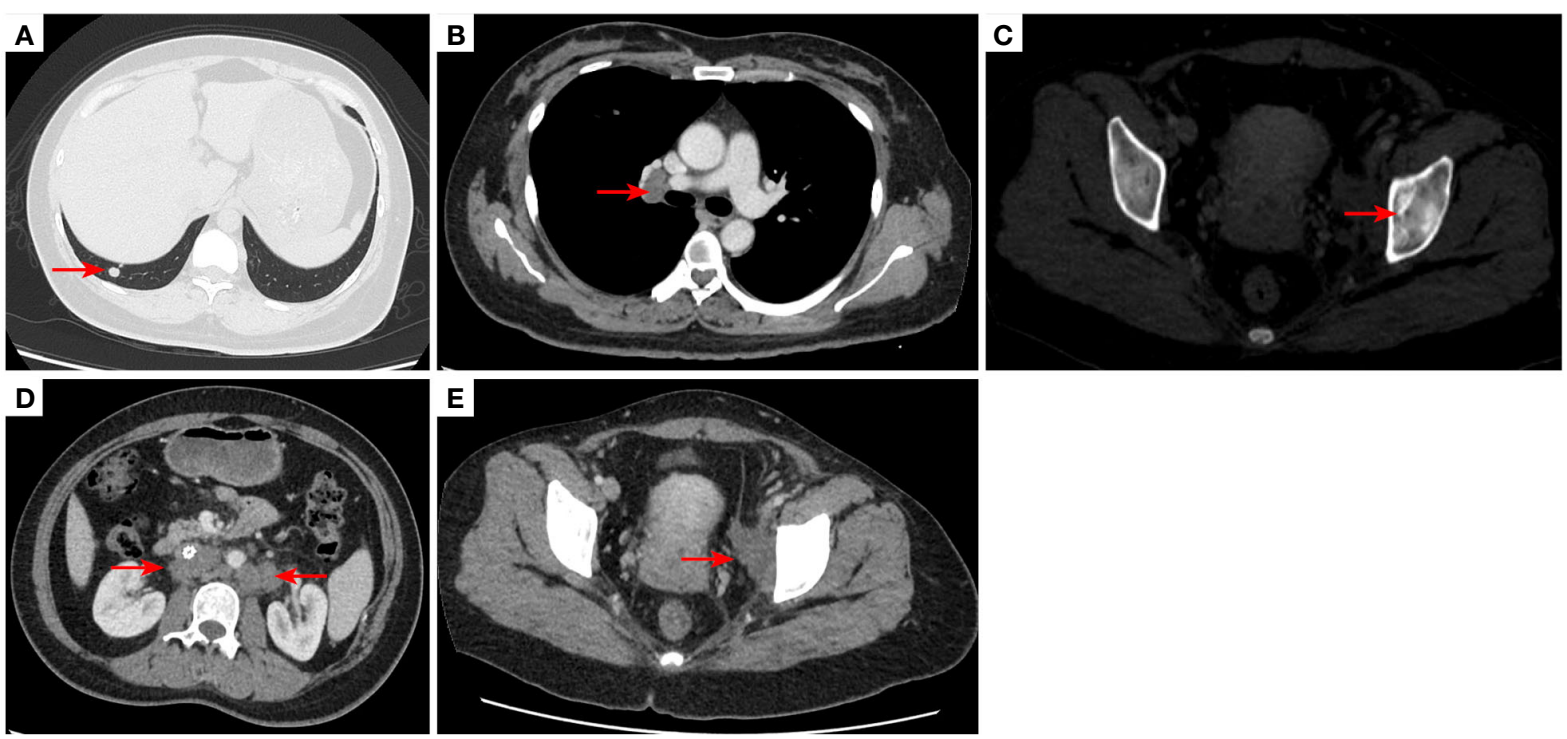
2020–12-22 CT scan: The right lower lobe of the lung metastatic tumor reappeared [red arrow, **(A)**] and the long diameter was 0.91 cm short diameter of right hilar lymph node was 1.66 cm [red arrow, **(B)**]; Left acetabular metastases [red arrow, **(C)**]. the short diameter of retroperitoneal lymph nodes was 1.66 cm, and the short diameter of left pelvic lymph nodes was 3.45 cm [red arrow, **(D, E)**]. The efficacy was PD. PD, progressive disease.

**Figure 6:**
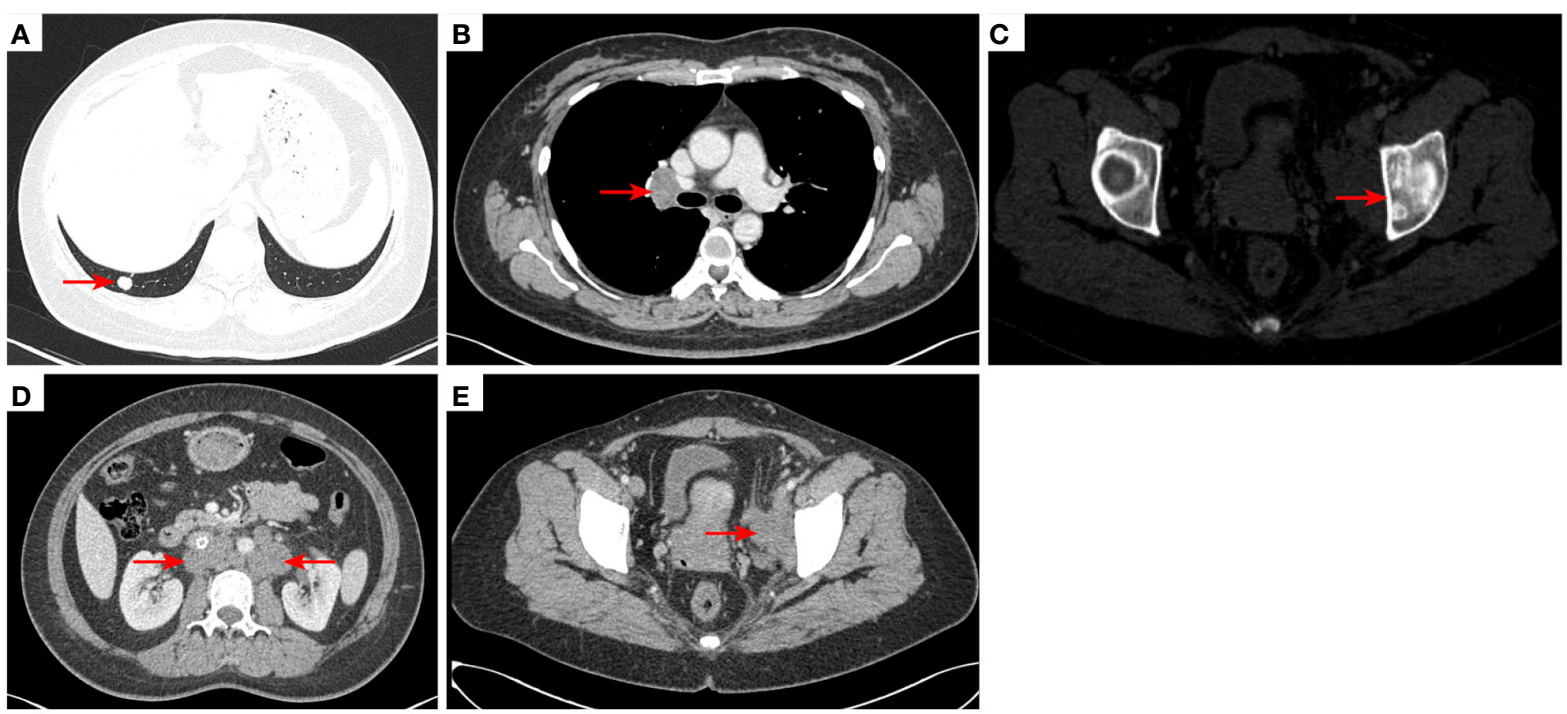
2021–2-3 CT scan: The right lower lung metastatic tumor was 1.28 cm [red arrow, **(A)**]; short diameter of right hilar lymph node 2.15 cm [red arrow, **(B)**]. Left acetabular metastases [red arrow, **(C)**]. The number of retroperitoneal lymph nodes increased and the short diameter was 1.61 cm [red arrow, **(D)**], and the left pelvic lymph nodes were 4.02 cm [red arrow, **(E)**]. The efficacy was PD. PD, progressive disease.

**Figure 7:**
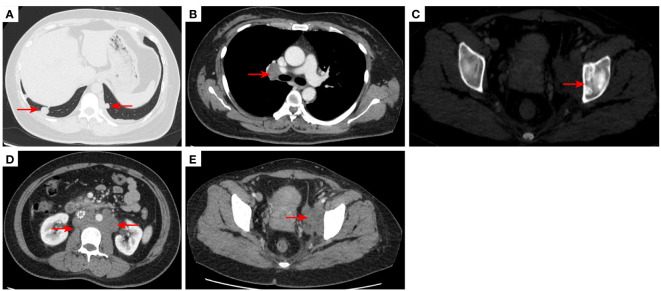
2021–3-9 CT scan: The number of lung metastases increased, and the length of the metastatic tumor in the right lower lobe of lung was 1.62 cm [red arrow, **(A)**], which was enlarged; short diameter of right hilar lymph node was 2.10 cm [red arrow, **(B)**]; Left acetabular metastases **(C)**. the short diameter of retroperitoneal lymph nodes was 2.00 cm, and the short diameter of left pelvic lymph nodes was 4.14 cm [red arrow, **(D, E)**]. The efficacy was unconfirmed progressive disease (iUPD) according irRECIST. irRECIST, immune-related response evaluation criteria in solid tumors.

**Figure 8:**
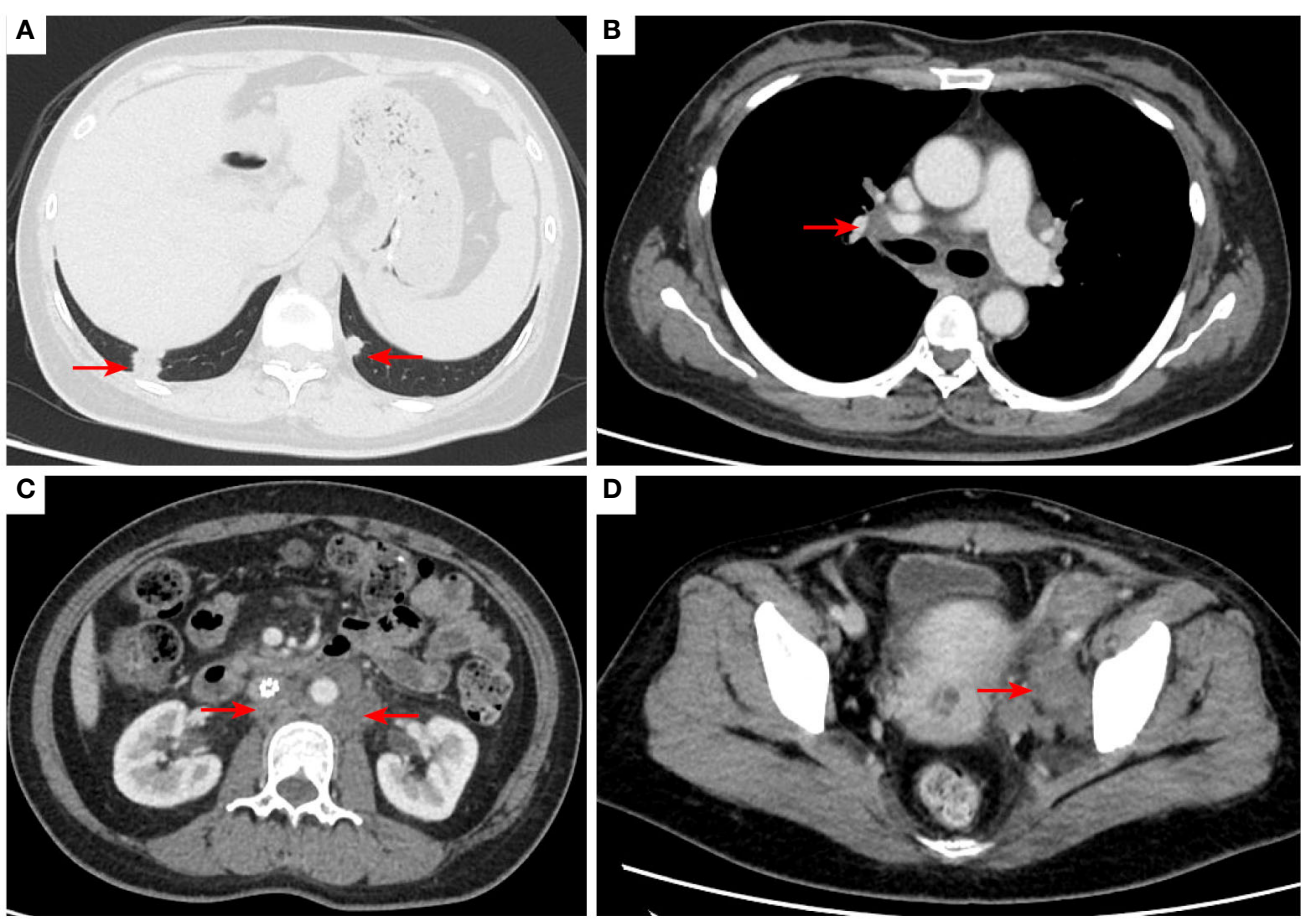
2021–8-24: Length of metastatic tumor in lower right lung 1.59 cm [red arrow, **(A)**]; short diameter of right hilar lymph node 1.53 cm [red arrow, **(B)**]; the short diameter of retroperitoneal lymph nodes was 1.61 cm, and the short diameter of left pelvic lymph nodes was 3.11 cm [red arrow, **(C, D)**]. The acetabulum was not scanned. The efficacy was SD. SD, stable disease.

**Figure 9:**
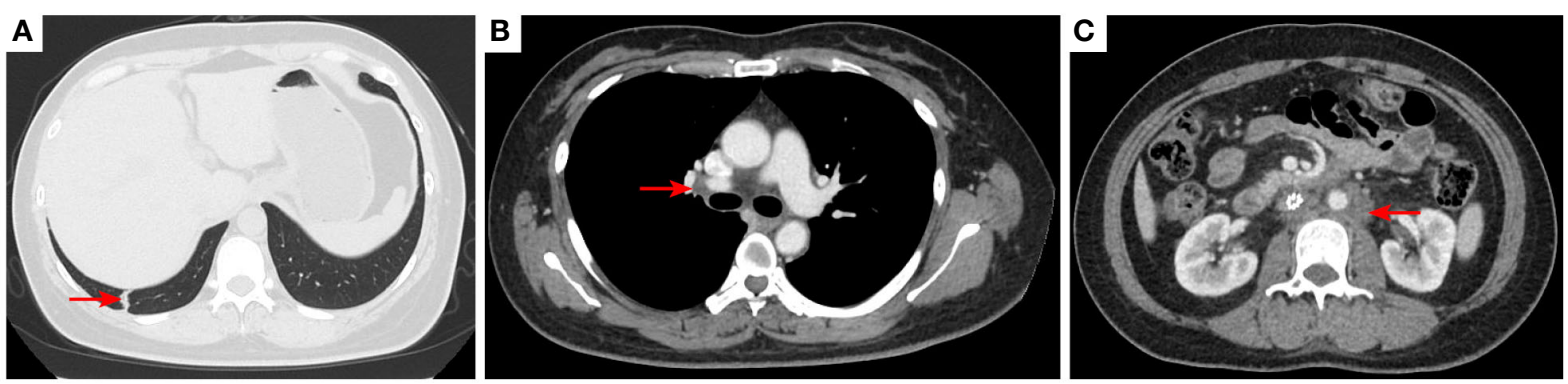
2021–12-15: Length of metastatic tumor in lower lobe of lung was 1.13 cm [red arrow, **(A)**]; short diameter of right hilar lymph node 1.26 cm [red arrow, **(B)**]; the short diameter of retroperitoneal lymph nodes was 1.56 cm [red arrow, **(C)**], and the pelvic cavity was not scanned. The efficacy was PR. PR, partial response.

**Figure 10:**
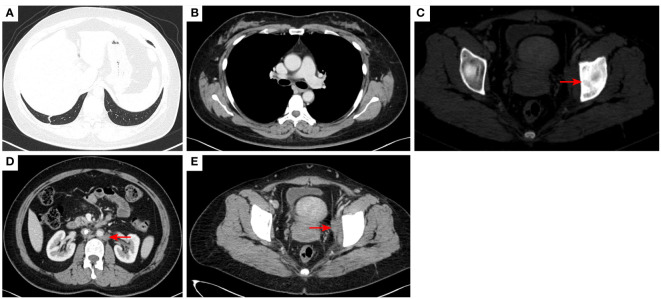
2023–4-13: The original right lower lobe of lung metastatic tumor disappeared **(A)**; The original right hilar enlargement lymph node disappeared **(B)**; Left acetabular metastases [red arrow, **(C)**]. The short diameter of retroperitoneal lymph nodes was 0.89cm [red arrow, **(D)**], and the short diameter of left pelvic lymph nodes was 2.13cm [red arrow, **(E)**].The efficacy was PR. PR, Partial response; SD, Stable disease; PD, Progressive disease; irRECIST, immune-related response evaluation criteria in solid tumors.

**Figure 11:**
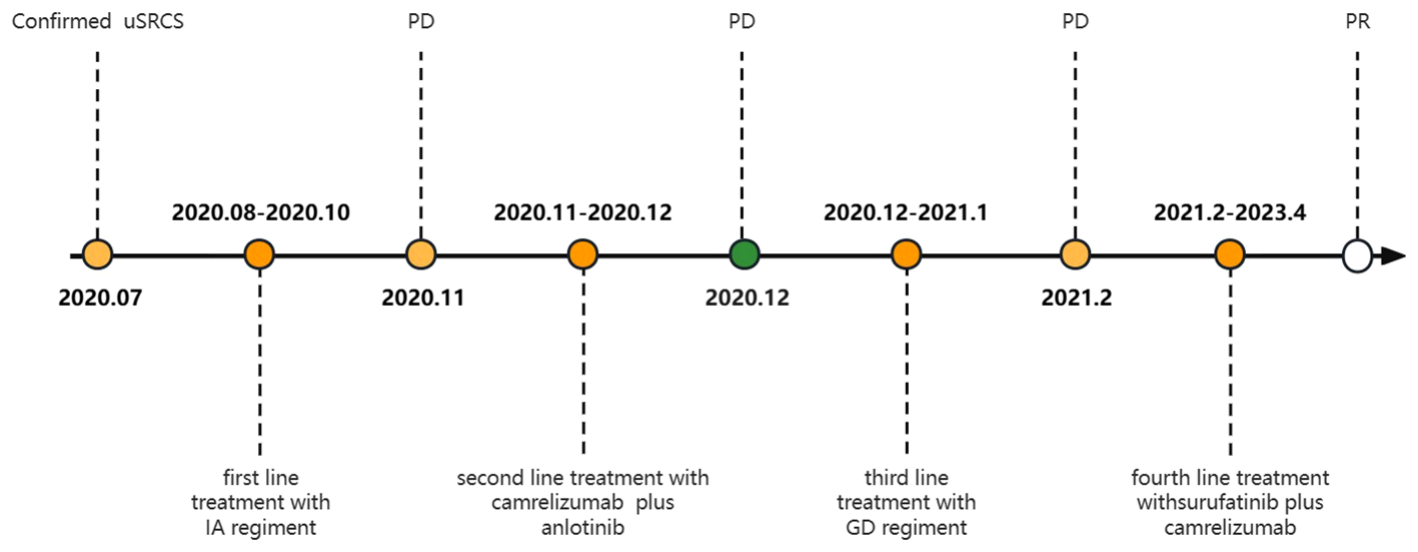
A timeline with relevant data from the episode of care.

The tumor stage was T4N1M1 with bone metastasis. The patient received 4 cycles of the IA regimen (ifosfamide 2g/m^2^, and epirubicin hydrochloride 30mg/m^2^ d1-d3) from August 5 to October 13, 2020. In September 2020, a CT examination revealed tumor shrinkage, which was consistent with stable disease. The pain, edema and claudication were relieved. On November 3, 2020, a CT scan revealed tumor regrowth. On November 4, 2020, the patient started 2 cycles of camrelizumab (a programmed death-1 inhibitor,200mg,q3w) plus anlotinib [an anti-vascular endothelial growth factor (VEGF) drug, 12mg,qd,2 weeks on/1 week off], but her disease progressed. Thus, a regimen of gemcitabine (900mg/m^2^, d1,d8,q3w) plus docetaxel (75mg/m^2^,d1,q3w) was administered on December 24, 2020 and January 14, 2021, but the tumor further progressed. Considering that the patient has already received third-line treatment and there is no standard treatment regimen, this patient can enter a clinical study or receive a new treatment. On February 4, 2021, after providing informed consent for experimental therapy, the patient began another line of targeted treatment with surufatinib(300mg,qd.po) combined with camrelizumab(200mg,q3w). A CT scan performed on December 24, 2021 showed a partial response. As of April 2023, the patient had a progression-free survival time of 26 months based on the last examination (**Figures 2**–**11**). No specific adverse effects were noted.

## 3 Discussion

Soft-tissue sarcomas comprise a broad range of tumors. Undifferentiated round-cell sarcomas are listed separately by the WHO (2020), and are high-grade and aggressive sarcomas with a poor prognosis. Using traditional immunohistochemical ancillary techniques, about 5% of all sarcomas remain unclassified (8). In this case, the patient was diagnosed with uSRCS by morphology, immunohistochemistry, NGS, and EWSR1/WT1 fusion gene studies. The morphology on pathological examination identified small round cells with atypical immunohistochemistry. As both CD99 and EWSR1-WT-1 fusion genes were negative, the diagnoses of desmoplastic small round-cell tumor and Ewing sarcoma were excluded. The current diagnostic criteria requires testing as many fusion genes as possible by RNA-sequencing assays or other fusion gene methods for classification (9). Both NGS of RNAseq testing on fusion gene were negative in our case.

Under current guidelines, the treatments of advanced sarcomas include chemotherapy and targeted therapies, but no specific treatment strategy is detailed for uSRCSs (10). The usual chemotherapy regimens include ifosfamide/anthracyclines-based regimens, taxanes, or gemcitabine-containing treatments (2, 11). Targeted therapy for sarcomas is largely dominated by small-molecule, multi-target, anti-vascular drugs, including pazopanib and anlotinib, which have been approved for the treatment of different types of sarcomas (5, 6). Small round-cell sarcomas are heterogeneous, and some are resistant, while other are sensitive to chemotherapy (12). In this case, the patient was resistant to both chemotherapy regimens used.

Anti-angiogenic multi-kinase inhibitors can be used in patients with chemo-resistant tumors. Due to the low incidence rate of uSRCSs, few patients with uSRCS have been included in clinical trials (13). Anlotinib is a novel tyrosine kinase inhibitor that targets multiple receptors and inhibits VEGF/vascular endothelial growth factor receptor (VEGFR) signaling by selectively targeting VEGFR-2, and -3 and fibroblast growth factor receptor (FGFR)-1, -2, -3, and -4 (11). Anlotinib was approved for the treatment of different types of sarcomas, but there were no uSRCSs in the clinical trial for the drug (14).

Surufatinib (Sulanda^®^ in China) is an oral, small-molecule, anti-VEGFR kinase inhibitor that targets VEGFR-1, -2, and -3, FGFR-1, and colony-stimulating factor-1 receptor (CSF-1R) (15) and was approved in China for the treatment of pancreatic and non-pancreatic neuroendocrine tumors (16). Soft-tissue sarcomas have a “cold” immune phenotype which is used to refer to inflamed but non T cell-infiltrated, or non-inflamed tumors, reflecting well the lower immunoscore categories (17). The use of immune checkpoint blockade in combination with anti-angiogenic drugs has a synergistic effect in cancer treatment and may overcome resistance to checkpoint inhibitors (18).

CSF-1R blockade by surufutinib may provide a therapeutic advantage compared with other multi-kinase inhibitors that do not block this receptor, as CSF-1 is associated with immune microenvironment (19). Macrophage infiltration has been identified as an independent poor prognostic factor in several cancer types. CSF-1 is a major survival factor for tumor infiltrating macrophages. In solid tumors, such as those of the breast and pancreas, infiltrating Cluster of differentiation 68 (CD68)+ or Cluster of differentiation 163 (CD163)+ tumor-associated macrophages are correlated with poor outcomes and immunosuppression (20).

Studies have reported a synergistic effect with anti-CSF1 and immunotherapy (19). The dual action of surufatinib in targeting tumor angiogenesis and CSF-1R might enhance its antitumor activity, while also making it suitable for use in combination with immune checkpoint inhibitors against various types of cancers. The synergistic effect between immune checkpoint blockades and anti-CSF-1 therapy may explain why the combination of surufatinib and immunotherapy was more effective than anlotinib plus immunotherapy in this patient. Other possible explanations for the effectiveness of surufatinib might include pharmacokinetics and pharmacodynamic potency in the inhibition of targeted kinases. This could result in the drug being effective in patients who are resistant to anlotinib (21).

No data related to sarcomas and uSRCS have been reported for surufatinib. This case report suggests that surufatinib in combination with checkpoint immunotherapy could be effective for uSRCS. The result of this study indicates that surufatinib should be explored for the treatment of sarcomas in prospective studies.

## Data availability statement

The datasets presented in this study can be found in online repositories. The names of the repository/repositories and accession number(s) can be found in the article/supplementary material.

## Ethics statement

The studies involving humans were approved by Ethics Committee of Guangdong Provincial Hospital of Chinese Medicine. The studies were conducted in accordance with the local legislation and institutional requirements. The participants provided their written informed consent to participate in this study. Written informed consent was obtained from the individual(s) for the publication of any potentially identifiable images or data included in this article.

## Author contributions

YL: Writing – original draft, Writing – review & editing. JH: Data curation, Writing – review & editing. XC: Investigation, Data curation, Writing – review & editing. YY: Investigation, Writing – original draft. XD: Investigation, Writing – review & editing. IV: Writing – review & editing. MS: Writing – review & editing. HZ: Writing – review & editing. ML: Supervision, Writing – review & editing.
